# Hindfoot Nailing for Ankle Fractures in High-Risk Elderly Patients: A Retrospective Analysis of Clinical and Radiological Outcome

**DOI:** 10.7759/cureus.96358

**Published:** 2025-11-08

**Authors:** Saif Abdulsattar, Shahid Mir, Chandan Vincent, Utkarsh Shahi, Mostafa Elfakhrany, Gopalkrishna G Verma, Vishal Kumar

**Affiliations:** 1 Trauma and Orthopedics, Manchester University NHS Foundation Trust, Manchester, GBR

**Keywords:** ankle fractures, distal tibia fractures, elderly patients, frailty, hindfoot nail, open fractures

## Abstract

Background

Hindfoot nailing (HFN) is an established surgical technique for ankle fractures, particularly in high-risk patients. Despite its advantages, outcomes can vary significantly based on patient factors and fracture characteristics.

Objective

The objective of this study is to evaluate short-term clinical and radiological outcomes of hindfoot nailing of ankle fractures in the frail elderly population, focusing on patient demographics, fracture characteristics, and complication rates to identify trends and areas for improvement.

Methods

A retrospective cohort study was conducted on 58 consecutive patients (mean age of 79.55 years; 47 (81%) women and 11 (19%) men) who underwent hindfoot nailing for ankle fractures between January 2020 and December 2024 at a tertiary trauma center. Patient demographics, fracture characteristics, and postoperative complications were analyzed. Primary outcomes included union rates and complication rates. Secondary outcomes included mortality, mobility changes, and the need for revision surgery. Statistical analysis employed Fisher's exact test and chi-square analysis to compare outcomes between open and closed fractures.

Results

The cohort demonstrated substantial frailty (mean Rockwood score of 5.38) and comorbidity burden (mean American Society of Anesthesiologists {ASA} grade of 3.10; 14 (24.6%) diabetics). Open fractures predominated (43, 63.3%). One-year mortality was 12 (20.7%) (95% confidence interval {CI}: 10.3%-31.1%). Among 30 patients with complete follow-up, the overall union rate was 24 (80.0%) (95% CI: 65.7%-94.3%), with comparable rates between open (16, 84.2%) and closed (nine, 81.8%) fractures (odds ratio {OR}: 1.19; p=0.986). However, open fractures demonstrated markedly elevated complication rates (12 {63.2%} versus two (18.2%); OR: 7.71; p=0.059). Joint preparation was associated with higher complication rates (11 {64.7%} versus three {23.1%}; OR: 6.11; p=0.077) but similar union rates. Functional mobility declined in 15 (50%) of patients, remained stable in 12 (40%), and improved in three (10%).

Conclusions

Hindfoot nailing in this high-risk population achieved acceptable union rates despite significant complication rates, particularly in open fractures. The findings support careful patient selection and highlight the need for enhanced perioperative protocols for open-fracture management.

## Introduction

Hindfoot nailing (HFN) has emerged as a valuable surgical technique for the management of complex fractures involving the distal tibia, ankle, and hindfoot regions [1,2]. This technique is particularly advantageous in elderly patients, those with compromised soft tissue envelopes, or cases where conventional fixation methods are not feasible [3]. The procedure offers several benefits, including stable fixation with minimal soft tissue disruption and the potential for early mobilization and primary definitive fixation with reduced total number of surgical procedures in such a frail, risky cohort. Moreover, acute shortening and primary hindfoot nailing can be a valid option for the definitive management of soft tissue coverage and fracture stabilization in a single procedure. Therefore, it might be an attractive option for these high-risk surgical candidates [4].

The aging population and the increasing prevalence of osteoporotic fractures have led to a growing need for surgical techniques that can provide reliable fixation in challenging clinical scenarios [5]. Hindfoot nailing addresses many of the limitations associated with traditional plate and screw fixation, particularly in patients with poor bone quality or extensive comminution [6]. The technique allows for load-sharing rather than load-bearing fixation, which can be crucial in maintaining stability during the healing process.

Despite these theoretical advantages, clinical outcomes following hindfoot nailing can vary considerably depending on multiple factors, including patient demographics, comorbidities, fracture characteristics, surgical technique, and postoperative care protocols [7]. Potential complications include infection, non-union, malalignment, and implant-related problems, which may significantly impact long-term function and the quality of life [8]. The complexity of managing these patients is further compounded by their often fragile physiological state and multiple comorbidities.

The continuous evaluation of surgical results is essential to ensure optimal patient care and to identify areas for improvement in clinical practice [9]. Understanding the specific challenges and outcomes associated with hindfoot nailing in high-risk populations is crucial for developing evidence-based treatment protocols and improving patient selection criteria.

The literature on hindfoot nailing outcomes has shown variable results, with some studies reporting excellent functional outcomes, while others highlight significant complication rates [10,11]. These discrepancies may be attributed to differences in patient populations, surgical techniques, and outcome measurement methods. Furthermore, many existing studies have focused on younger, healthier populations, leaving a knowledge gap regarding outcomes in elderly, frail patients who increasingly represent the typical hindfoot nailing candidate.

The aim of this study was to evaluate the clinical and radiological short-term outcomes of hindfoot nail surgeries for ankle fractures in the elderly, frail population performed at a major trauma center, with a specific focus on patient demographics, fracture characteristics, and complication rates. By analyzing these factors comprehensively, we sought to identify trends, benchmark our results against published standards, and highlight areas for improvement in clinical practice.

## Materials and methods

Study design and setting

This retrospective cohort study was conducted at a single tertiary referral center and level I trauma facility serving a population of approximately 2.8 million. The study protocol was registered with the Manchester University NHS Foundation Trust Clinical Governance Department and approved as a service evaluation initiative (registration number: S467). The investigation was conducted in accordance with institutional audit governance protocols and adhered to Strengthening the Reporting of Observational Studies in Epidemiology (STROBE) guidelines for observational research. All patient data were de-identified prior to analysis to ensure confidentiality and compliance with data protection standards.

Patient population

A consecutive series of 58 patients who underwent hindfoot nailing for ankle or distal tibia fractures between January 2020 and December 2024 were identified through institutional electronic health records (Hive Electronic Patient Record system). All patients treated with hindfoot nailing during this period were included regardless of age, medical comorbidities, fracture configuration, or open versus closed injury status. This inclusive approach was deliberately chosen to capture the full spectrum of clinical complexity encountered in real-world practice and to avoid selection bias that might artificially improve reported outcomes. No exclusion criteria were applied.

Surgical technique and implant system

All procedures utilized the Orthofix hindfoot nailing system, reflecting institutional preference and surgeon familiarity. Surgical technique followed manufacturer recommendations with patient-specific modifications based on fracture configuration and soft tissue status. Briefly, patients were positioned supine with the operative extremity prepared and draped in standard sterile fashion. A percutaneous approach through the plantar hindfoot facilitated guide wire insertion under fluoroscopic guidance, with sequential reaming, followed by nail insertion and proximal/distal interlocking screw fixation.

In cases presenting with severe soft tissue compromise, external fixation was utilized as a temporizing measure (damage control orthopedics approach) to permit wound stabilization and edema resolution prior to definitive nailing. Joint preparation, defined as deliberate articular cartilage debridement to create tibiotalocalcaneal arthrodesis, was performed selectively based on articular surface damage, fracture comminution involving the tibiotalar or subtalar joints, and the intraoperative assessment of fracture stability without fusion.

Soft tissue management strategies were individualized according to wound characteristics, ranging from primary closure when feasible to delayed closure, split-thickness skin grafting, or local/regional flap coverage for complex defects. All patients received protocol-driven antibiotic prophylaxis and tetanus prophylaxis as appropriate for injury type.

Postoperative care followed standard institutional protocols for wound care and early mobilization, but no additional standardized rehabilitation regimen beyond usual practice was implemented.

Data collection

Comprehensive clinical data were systematically extracted from electronic medical records by two independent reviewers. Patient demographics included age, sex, body mass index (BMI), and the presence of diabetes mellitus. Frailty assessment utilized the American Society of Anesthesiologists (ASA) physical status classification and the validated Rockwood Clinical Frailty Scale (scores 1-9, with higher scores indicating greater frailty) [12].

Fracture characteristics documented included anatomical location (ankle versus distal tibia) and injury classification (open versus closed). Open fractures were defined per standard criteria as any fracture with communication between the fracture site and the external environment.

Surgical variables recorded encompassed the use of temporary external fixation, the performance of joint preparation, and the soft tissue closure technique. Postoperative follow-up data captured both surgical complications (infection, non-union, malunion, implant failure, and wound healing problems) and medical complications (cardiac events, respiratory complications, and thromboembolic events). Union was determined radiographically at routine follow-up visits (typically 3-4 months postoperatively) based on bridging callus across ≥3 cortices and the clinical assessment of pain-free weight-bearing.

Functional outcomes were assessed by comparing pre-injury with post-injury mobility status, categorized as improved, unchanged, or declined.

Statistical analysis

Descriptive statistics were calculated for all variables. Continuous variables were summarized using means, medians, standard deviations, and ranges. Categorical variables were presented as frequencies and percentages. The normal distribution of continuous variables was assessed using the Shapiro-Wilk test.

Comparative analyses between groups (open versus closed fractures and with versus without joint preparation) employed Fisher's exact test for categorical variables due to small sample sizes in some cells. Odds ratios (OR) with 95% confidence intervals (CI) were calculated to quantify the strength of associations between risk factors and outcomes. Proportions were reported with 95% confidence intervals calculated using the Wilson score method. Statistical significance was defined as p<0.05 (two-tailed). All statistical analyses were performed using SPSS version 28.0 (IBM Corp., Armonk, NY).

## Results

Patient demographics and baseline characteristics

The study cohort comprised 58 patients with demographic and clinical characteristics detailed in Table 1. The population was markedly elderly (mean age of 79.55 years; range: 46-100) with pronounced female predominance at 47 (81%) and 11 (19%) men, consistent with established epidemiological patterns of fragility fractures [13]. The majority of patients were octogenarians or older, reflecting the typical demographic receiving hindfoot nailing in contemporary practice.

**Table 1:** Patient Demographics and Baseline Characteristics (N=58) SD, standard deviation; ASA, American Society of Anesthesiologists

Characteristic	Value
Age (Years), Mean±SD	79.55±12.00
Age (Years), Median (Range)	80.0 (46-100)
Sex: Female, n (%)	47 (81.0%)
Sex: Male, n (%)	11 (19.0%)
Body Mass Index (kg/m²), Mean±SD	31.61±8.50
Body Mass Index (kg/m²), Median (Range)	30.90 (17.70-53.40)
Diabetes Mellitus, n (%)	14 (24.6%)
Rockwood Frailty Score, Mean±SD	5.38±1.57
Rockwood Frailty Score, Median (Range)	6.0 (2-8)
ASA Grade, Mean±SD	3.10±0.63
ASA Grade, Median (Range)	3.0 (2-4)
ASA 3 or 4, n (%)	49 (84.5%)

Body mass index data revealed a mean BMI of 31.61 (median, 30.90; range, 17.70-53.40; standard deviation, 8.50), indicating that the majority of patients were overweight or obese. Diabetes mellitus was present in a significant proportion of the cohort (14/58 patients, 24.6%), representing an important comorbidity that could influence both surgical outcomes and healing potential.

Frailty and comorbidity assessment

**Figure 1:**
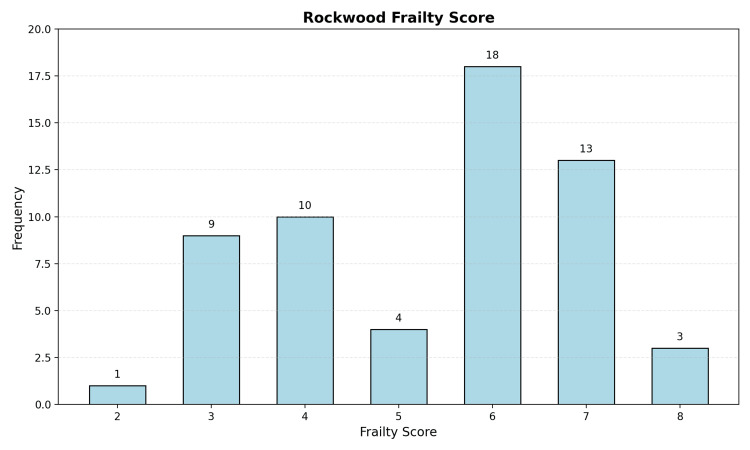
Rockwood Frailty Score by Frequency of Patients (N=58)

The Rockwood Frailty Score analysis revealed a high-risk population with a mean score of 5.38 (median, 6.0; range, 2-8; standard deviation, 1.57). The distribution showed a predominance of scores between 6 and 7 (Figure 1), indicating that most patients were moderately to severely frail. The ASA grade distribution further confirmed the high-risk nature of this population, with a mean ASA grade of 3.10 (median, 3.0; range, 2-4; standard deviation, 0.63). Forty-nine patients (84.5%) were classified as grade 3 or 4, denoting severe systemic disease with or without functional limitation.

Fracture characteristics

**Figure 2:**
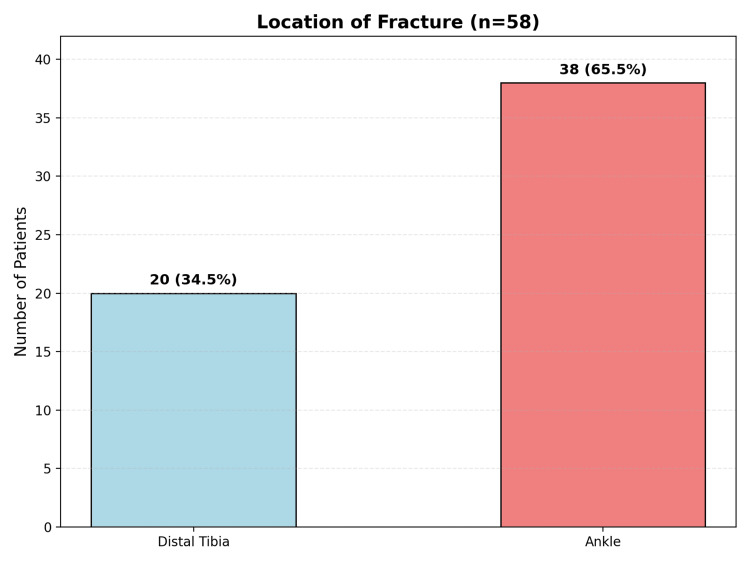
Number of Patients by the Location of Fracture

The analysis of fracture location revealed that ankle fractures were significantly more common than distal tibia fractures, representing 38 patients versus 20 for the distal tibia population (Figure 2). The fracture type distribution showed a predominance of open fractures, which has important implications for infection risk, surgical planning, and postoperative care. Open fractures comprised 43 (63.3%) cases, while closed fractures accounted for 15 cases (36.7%).

Surgical interventions

Hindfoot nailing constituted the definitive fixation method in all cases, with the Orthofix system employed in 100% of procedures, reflecting institutional preference and surgeon familiarity with this particular implant system. External fixation was utilized as a temporizing intervention in 21 (36.2%) cases with severe soft tissue compromise, permitting wound stabilization and edema resolution prior to definitive nailing, consistent with damage control orthopedics principles [14].

Joint preparation, defined as deliberate articular cartilage debridement to create tibiotalocalcaneal arthrodesis, was performed in 26 (44.8%) patients. The decision to perform joint preparation was predicated on the extent of articular surface damage, comminution involving tibiotalar or subtalar articulations, and the operating surgeon's assessment regarding the achievability of stable fracture reduction without arthrodesis [15].

Soft tissue management strategies varied according to injury severity, with primary wound closure achieved in 37 (63.79%) cases, while more sophisticated reconstructive procedures requiring split-thickness skin grafting (two patients) or flap coverage (four patients) proved necessary in 10.34% of cases.

Follow-up analysis

At one-year follow-up, mortality was 12 patients (20.7%; 95% CI: 10.3%-31.1%). Among the survivors, 16 patients had no follow-up data available, leaving 30 patients (51.7% of the original cohort) for detailed outcome analysis.

Detailed analysis of 30 patients with complete follow-up

Among the 30 patients with complete follow-up data, 19 (63.3%) presented with open fractures, while 11 (36.7%) had closed fractures. The overall fracture union rate was 80.0% (24/30 patients; 95% CI: 65.7%-94.3%). Notably, union rates did not differ significantly between open and closed fractures (84.2% versus 81.8%; OR, 1.19; p=0.986), suggesting that the biological healing capacity was not substantially impaired by the open nature of the injury when appropriate surgical management was provided (Table 2).

**Table 2:** Clinical Outcomes Stratified by Fracture Type (N=30 With Complete Follow-Up) Fisher's exact test used for statistical comparison CI, confidence interval; CRPS, complex regional pain syndrome

Outcome	Open Fractures (n=19)	Closed Fractures (n=11)	Odds Ratio (95% CI)	P-value
Union, n (%)	16 (84.2%)	9 (81.8%)	1.19 (0.15-9.37)	0.986
95% CI for Proportion	65.5%-100%	57.5%-100%	-	-
Complications, n (%)	12 (63.2%)	2 (18.2%)	7.71 (1.31-45.5)	0.059
95% CI for Proportion	41.5%-84.8%	0%-41.0%	-	-
Specific Complications				
Wound Healing Problems, n (%)	3 (15.8%)	0 (0%)	-	-
Infection, n (%)	2 (10.6%)	1 (9.1%)	-	-
Implant-Related, n (%)	2 (10.6%)	1 (9.1%)	-	-
Amputation, n (%)	1 (5.3%)	0 (0%)	-	-
Nerve Injury, n (%)	1 (5.3%)	0 (0%)	-	-
CRPS, n (%)	1 (5.3%)	0 (0%)	-	-
Periprosthetic Fracture, n (%)	1 (5.3%)	0 (0%)	-	-

In stark contrast to the comparable union rates, complication rates differed markedly by fracture type. Open fractures demonstrated substantially higher overall complication rates compared to closed fractures (63.2% versus 18.2%; OR, 7.71; p=0.059), approaching but not quite reaching statistical significance at the conventional α=0.05 threshold. This near-significant finding nonetheless represents a clinically meaningful nearly 3.5-fold increase in complications among open injuries, with the limited sample size likely preventing the achievement of formal statistical significance.

Specific complication analysis

Among the 19 patients with open fractures, seven (36.8%) experienced no complications. The remaining 12 patients (63.2%) encountered various issues, including complex wound healing in three patients (15.8%), revision due to prominent nail in one patient (5.3%), and screw back-out in another one patient (5.3%). Metalwork infection was reported in two patients (10.6%), with one requiring revision surgery and the other managed with debridement and washout. Additional complications included complex regional pain syndrome in one patient (5.3%), periprosthetic fracture in one patient (5.3%), and prosthetic left talus implant infection leading to below-knee amputation in one patient (5.3%). Lastly, nerve damage involving the superficial peroneal nerve was observed in one patient (5.3%).

Among the 11 patients with closed fractures, nine (81.8%) experienced no complications. However, two patients (18.2%) did encounter issues: One patient (9.1%) developed a metalwork infection that required revision surgery, and another patient (9.1%) experienced implant loosening.

Effect of joint preparation

Joint preparation was performed in 17 patients, accounting for 56.7% of the cohort. Out of the 17 patients who had joint preparation, 14 of those had open injuries, and three patients had joint preparation and closed fractures. Patients undergoing joint preparation demonstrated higher complication rates (11 {64.7%} versus three {23.1%}; OR, 6.11; p=0.077) but lower union rates (13 {76.5%} versus 12 {92.3%}; OR, 0.27; p=0.514) compared to those without joint preparation, though neither comparison achieved statistical significance (Table 3). The elevated complication rate in this subgroup probably reflects the baseline injury severity rather than any iatrogenic effect of the joint preparation itself. Of the 17 patients who underwent joint preparation, 14 (82.4%) had open fractures, further supporting the interpretation that joint preparation served as a marker for injury severity.

**Table 3:** Clinical Outcomes Stratified by Joint Preparation Status (N=30) Fisher's exact test used for statistical comparison CI: confidence interval

Outcome	Joint Preparation (n=17)	No Joint Preparation (n=13)	Odds Ratio (95% CI)	P-value
Union, n (%)	13 (76.5%)	12 (92.3%)	0.27 (0.03-2.79)	0.514
Complications, n (%)	11 (64.7%)	3 (23.1%)	6.11 (1.22-30.5)	0.077
Open Fractures, n (%)	14 (82.4%)	5 (38.5%)	7.47 (1.48-37.8)	0.022
Mobility at Follow-up				
Improved, n (%)	1 (5.9%)	2 (15.4%)	-	-
Unchanged, n (%)	7 (41.2%)	5 (38.5%)	-	-
Declined, n (%)	9 (52.9%)	6 (46.2%)	-	-

Functional outcomes

Functional outcomes were assessed based on patients' ability to return to their pre-injury level of mobility. Of the 30 patients with follow-up data, mobility had declined in 15 patients (50.0%), remained at baseline levels in 12 patients (40.0%), and improved in three patients (10.0%) (Table 3).

## Discussion

This study provides valuable insights into the outcomes of hindfoot nailing in a high-risk, predominantly elderly and frail patient population. The findings demonstrate both the potential benefits and significant challenges associated with this surgical technique in complex clinical scenarios.

The demographic profile of our cohort reflects the typical population requiring hindfoot nailing, with a mean age approaching 80 years and high levels of frailty and comorbidity [12]. The predominance of ASA grade 3 patients and high Rockwood Frailty Scores (mean: 5.38) indicates that these procedures are being performed in patients with significant physiological compromise [16]. This patient profile reflects the growing trend of preferring HFN in elderly patients who, in the past, might have been considered unsuitable for surgical intervention. This is due to the merits of being a minimally invasive procedure, having a shorter operative time, and less need for staged procedures [17].

The high prevalence of obesity in our cohort (mean BMI: 31.61) adds another layer of complexity to surgical management, as elevated BMI has been associated with increased complication rates in orthopedic surgery, particularly regarding wound healing and infection [18]. The combination of advanced age, frailty, and obesity creates a challenging surgical environment that requires careful perioperative planning and risk stratification.

The predominance of open fractures (63.3%) in our series is notable and likely reflects both the high-energy nature of many of these injuries and the referral pattern to our institution as a tertiary care center [19]. Open fractures present unique challenges in terms of infection risk, soft tissue management, and healing potential, which are clearly reflected in our complication data.

The overall union rate of 80% in our series is encouraging, particularly given the high-risk nature of the patient population. Interestingly, open and closed fractures demonstrated similar union rates (84.2% versus 81.8%), suggesting that the biological healing potential may not be significantly compromised by the open nature of the fracture when appropriate surgical management is employed [20].

These union rates compare favorably with published literature on hindfoot nailing, where union rates typically range from 75% to 95% depending on patient selection and fracture complexity [21]. The similarity in union rates between open and closed fractures may be attributed to the load-sharing nature of intramedullary fixation, which provides mechanical stability while allowing biological healing to proceed, and avoiding the disadvantage of adding metalwork, which might hinder soft tissue healing.

The most striking finding of our analysis is the marked disparity in complication rates between open and closed fractures (12 {63.2%} versus two {18.2%}; OR, 7.71; p=0.059). While this comparison narrowly missed achieving statistical significance (p=0.059), the magnitude of the difference, a nearly eightfold increased odds of complications in open fractures, represents a clinically critical finding that should inform both treatment decisions and informed consent discussions [10,19]. The failure to achieve formal statistical significance (p<0.05) likely reflects insufficient statistical power given our sample size rather than the absence of a true clinical effect. The calculation of the required sample size suggests that approximately 40-50 patients per group would be needed to achieve 80% power to detect this magnitude of difference with α=0.05.

The most common complication in open fractures was complex wound healing (15.8%), which reflects the challenging soft tissue environment often encountered in these cases. The presence of severe complications such as below-knee amputation (5.3%) and permanent nerve damage (5.3%) exclusively in the open fracture group underscores the potential for devastating outcomes in this subset of patients.

Infection-related complications occurred three times more frequently in open fractures compared to closed fractures, which aligns with established risk factors for surgical site infection in orthopedic surgery [10,18,19]. The spectrum of infection severity ranged from cases manageable with debridement and washout to those requiring amputation, highlighting the importance of aggressive infection prevention and the early recognition of complications. Strategies to reduce infection risk include meticulous and timely debridement, the adoption of an orthoplastic approach with early soft tissue coverage, the use of local antibiotic carriers such as Stimulan, the application of negative pressure wound dressings, and the close involvement of a dedicated complex wound care team [22].

The findings that patients requiring joint preparation had higher complication rates (64.7% versus 23.1%) but lower union rates (76.5% versus 92.3%) likely reflect selection bias, as joint preparation is typically reserved for more complex cases with greater articular involvement. These patients often present with more severe initial injuries and compromised bone quality or require more extensive surgical procedures, all of which contribute to increased complication risk. In our series, the joint was not routinely prepared, a practice supported by the literature, which suggests that satisfactory outcomes can be achieved without formal joint preparation in most cases. Recent evidence indicates that retrograde hindfoot nailing provides stable fixation and acceptable union rates in elderly or medically fragile patients without the need for additional joint preparation, thereby minimizing operative time and soft tissue insult [1,2,17].

The decision to perform joint preparation is usually based on the degree of articular damage and the likelihood of developing post-traumatic arthritis [23]. While this may increase immediate surgical complexity and complication risk, it may be necessary to optimize long-term functional outcomes and prevent the need for future revision surgery.

The mobility outcomes reveal that 50% of patients experienced a decline from their baseline functional status, while 40% maintained their pre-injury level, and only 10% showed improvement. Rather than quantifying the degree of mobility loss, which is not practical in this population, functional recovery is more appropriately judged by the ability to return to previous levels of activity. This pattern is not unexpected given the advanced age and frailty of the cohort, as well as the significant nature of the injuries treated. Optimizing functional outcomes requires a proactive approach, including early mobilization where feasible and structured physiotherapy programs, both of which have been shown to enhance recovery and support return to activity in older adults following ankle fracture [9,24].

The high rate of functional decline emphasizes the importance of realistic goal-setting and comprehensive rehabilitation planning in this patient population [24]. The primary goal in many cases may be the maintenance of independence and the prevention of further deterioration rather than restoration to pre-injury function.

The 20% one-year mortality rate, while concerning, must be interpreted in the context of the patient population's baseline risk profile. Importantly, none of the deaths in our study were acute or occurred in the immediate postoperative period, suggesting that mortality was more reflective of underlying frailty and comorbidities rather than the surgical intervention itself. Studies of elderly patients with hip fractures, a comparable population in terms of age and frailty, report similar or higher mortality rates [25].

Furthermore, published series of elderly patients treated with hindfoot nails for fragility ankle fractures report one-year mortality rates ranging from 25% to 46%, again highlighting that these outcomes are largely driven by patient factors rather than fixation method [2,17]. The high prevalence of significant comorbidities and frailty in our cohort likely contributes to this mortality rate, which may not be directly attributable to the surgical intervention itself.

Clinical implications and quality improvement

Several important clinical implications emerge from this analysis. The high complication rate in open fractures underscores the need for enhanced patient selection criteria and robust risk stratification protocols [26]. Given the high prevalence of medical comorbidities in this cohort, comprehensive perioperative medical optimization is essential to reduce adverse outcomes [27]. The significant infection burden associated with open fractures further highlights the importance of rigorous infection prevention strategies, including timely antibiotic prophylaxis and meticulous soft tissue management [28]. In addition, the high rate of functional decline emphasizes the necessity of early and comprehensive rehabilitation planning, with consideration for prehabilitation strategies to maximize recovery potential. Finally, the complex nature of these patients requires coordinated multidisciplinary care, involving orthopedic surgeons, geriatricians, physiotherapists, and other specialists, to ensure holistic management and optimize both short- and long-term outcomes.

Limitations

Several limitations must be acknowledged in this study. The retrospective design limits the ability to control for confounding variables and may introduce selection bias. The relatively small sample size, particularly after accounting for mortality and loss to follow-up, may limit the generalizability of findings. The single-center design may not reflect outcomes at other institutions with different patient populations or surgical techniques.

The lack of standardized functional outcome measures limits the ability to compare results to other studies and may not fully capture the impact of surgery on patient quality of life. Additionally, the follow-up period may not be sufficient to assess long-term complications such as post-traumatic arthritis or implant failure.

Future directions

Future research should focus on developing risk stratification tools to better identify patients who are most likely to benefit from hindfoot nailing while minimizing complications. Prospective studies with standardized outcome measures and longer follow-up periods would provide more robust evidence for clinical decision-making [29].

The investigation of specific techniques for managing open fractures, including the optimal timing of definitive fixation and soft tissue reconstruction strategies, could help reduce the high complication rates observed in this subset of patients. Additionally, research into perioperative optimization protocols and enhanced recovery pathways may help improve outcomes in this high-risk population [30].

## Conclusions

In light of our study, we believe that hindfoot nailing represents a valid surgical option for ankle fractures in patients with complex frailty, multiple comorbidities, and high ASA grades. Its minimally invasive nature preserves soft tissue integrity, while the reduced operative time offers a particular advantage in anesthetically high-risk patients. Furthermore, it is a versatile technique that allows for acute primary shortening, providing a single surgical procedure to address both bony and soft tissue challenges simultaneously. Despite achieving acceptable union rates, the high complication and mortality rates in this frail cohort underscore the need for individualized surgical decision-making and comprehensive multidisciplinary perioperative care.
